# Robust natural depth for anticorrelated random dot stereogram for edge stimuli, but minimal reversed depth for embedded circular stimuli, irrespective of eccentricity

**DOI:** 10.1371/journal.pone.0274566

**Published:** 2022-09-22

**Authors:** Paul B. Hibbard, Jordi M. Asher

**Affiliations:** Department of Psychology, University of Essex, Colchester, Essex, United Kingdom; Justus Liebig Universitat Giessen, GERMANY

## Abstract

The small differences between the images formed in our left and right eyes are an important cue to the three-dimensional structure of scenes. These disparities are encoded by binocular neurons in the visual cortex. At the earliest stage of processing, these respond to binocular correlation between images. We assessed the perception of depth in anticorrelated stimuli, in which the contrast polarity in one eye is reversed, as a function of their location in the retinal image, and their depth configuration (a horizontal edge or a circle surrounded by an annulus) We found that, regardless of stimulus eccentricity, participants perceived depth in the natural direction for edge stimuli, and weakened, reversed depth for circular stimuli.

## Introduction

### Binocular vision and binocular disparity

**Fig 1:**
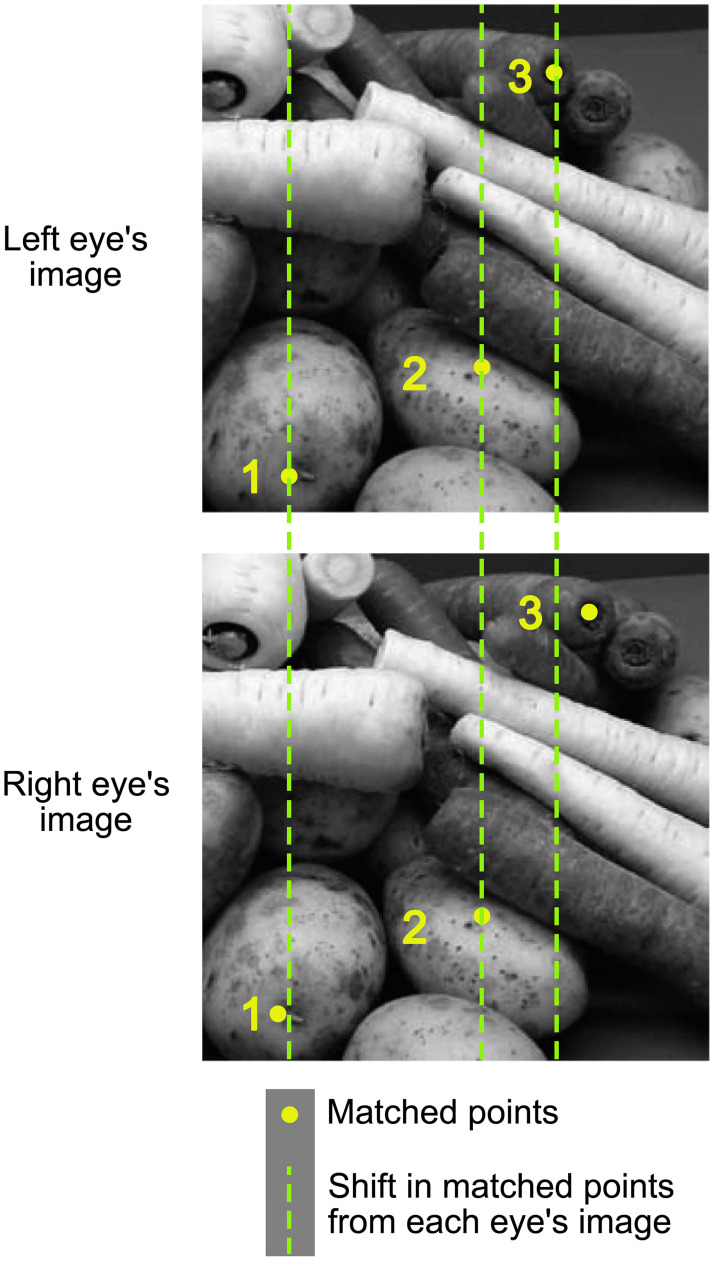
Each eye, the left (top) and right (bottom), receives a different image depending on fixation (point 2). The point of fixation falls on the same retinal location and has zero disparity. For point **1**, there is a small shift leftwards in the right eye’s image in comparison to point **1** in the left eye’s image. This *crossed* disparity arises as this point in the image is closer than the fixation point. Conversely, there is a rightward shift of point **3** from the left to the right eye’s image. This *uncrossed* disparity arises as this point is further away than the fixation point. Point **1** will appear closer and **3** further away. Images are drawn from [2].

The perception of depth from binocular vision depends on binocular disparities, the differences between the left and right eyes’ images. These disparities are encoded in the primary visual cortex by binocular neurons that have a receptive field for each eye (Fig 1). Receptive fields are combined in such a way that binocular simple and complex cells in V1 have a characteristic disparity tuning function—that is, the strength of their responses depends on the disparity of the stimulus presented within their receptive fields [1].

Disparity-tuned neurons in the primary visual cortex form the early stages of a broadly distributed network of binocular processing. At higher levels of visual processing, binocular responses are found in both the dorsal and ventral streams [3]. This broad distribution of binocular processing reflects the importance of three-dimensional information for both action and perception, and demonstrates the complexity of extracting depth from binocular signals.

### Modelling binocular vision

**Fig 2:**
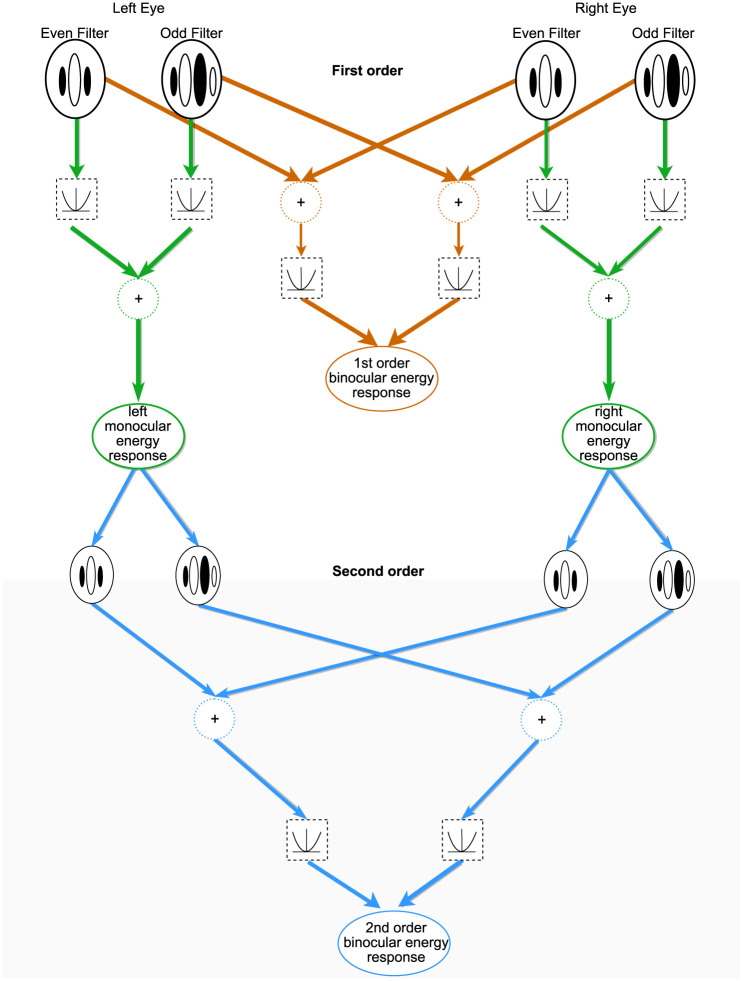
Outline of a first order binocular energy model (orange pathway): Odd and even symmetric monocular receptive fields (white areas represent excitatory regions of the receptive fields and dark areas inhibitory regions) are combined to compute the first order binocular energy response. Responses are summed across the two eyes for corresponding filters, then squared. The squared outputs are summed across the two halves of the quadrature pair. Outline of a second order binocular energy model (green and blue pathway): Odd and even monocular energy responses are calculated separately for each eye (green pathway). The first-order responses are squared and summed, to produce monocular energy responses for each eye. This forms the input to the second order filters (blue pathway) where the pairs of monocular filters are summed, squared and combined to calculate the second-order binocular energy response.

The binocular energy model (Fig 2) gives a computational account of the responses of disparity-tuned binocular neurons in the primary visual cortex [4, 5]. In this model, odd- and even-symmetric Gabor functions are used to model the binocular receptive fields of neurons. These first-stage filters sum inputs from the left- and right-eyes’ images; the responses of these quadrature pairs of linear first-stage binocular filters are then squared and combined to produce responses that are sensitive to the binocular disparities of features, but invariant to the locations of these features within the receptive field. Disparity tuning in this model depends on differences in the position and structure (phase) of the receptive fields between the left and right eye.

**Fig 3:**
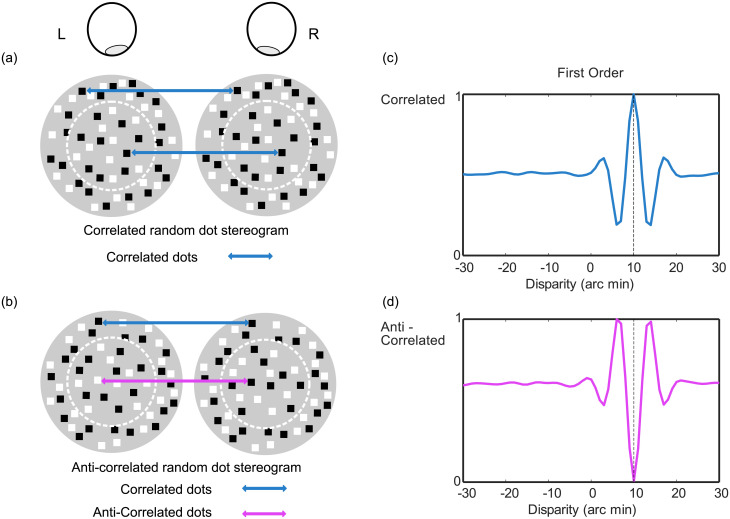
**(a)** Image pair for a correlated random dot stereogram, where the outer annulus and the inner circle (the detection target) contain correlated (matching) dots in the images shown to the left and right eyes. **(b)** Image pair for an anticorrelated random dot stereogram, where the outer annulus contains correlated (matching) dots in the images shown to the left and right eyes. In contrast to the correlated target in (a), the target circle contains anticorrelated (reversed polarity) dots in the images shown to the left and right eye. **(c)** an example first order binocular energy tuning function in response to a correlated random dot stereogram. This tuning function has a preferred disparity at 10 arc minutes **(d)** the same first order binocular energy tuning function in response to an anticorrelated random dot stereogram. The tuning function is inverted and now has a preferred disparity at 5 arc minutes.

Our understanding of how populations of neurons modelled in this way give rise to the perception of depth has been greatly enhanced by the use of random dot stereograms (RDS) [6]. These consist of a stereo-pair of patterns of randomly positioned dots and allow us to isolate binocular cues to depth in a laboratory setting. In a classic, correlated random dot stereogram (CRDS), the positions of some of these dots will be shifted between the two eyes to create non-zero disparities (Fig 3a), which are either ‘crossed’ (consistent with near depth) or ‘uncrossed’ (consistent with far depth). Humans and many other species perceive depth consistent with the disparities present in the stimuli. Binocular cells in the visual cortex respond to CRDS with clearly-defined disparity tuning [7–10].

A variation on these stimuli, known as anticorrelated random dot stereograms (ACRDS), can be used to test models of binocular vision. In an ACRDS, the contrast polarity of elements is reversed between the two eyes, such that bright pixels in the left eye are dark in the right eye, and vice versa (Fig 3b). The binocular energy model predicts that the disparity tuning function should be inverted in ACRDS in comparison with CRDS. This means that the preferred and anti-preferred disparities (the stimulus disparities creating the largest, and smallest, responses respectively) are switched, and thus a different stimulus disparity is required to produce the maximum response for correlated and anticorrelated stimuli (see Fig 3c & 3d respectively). The disparity predicted to give the largest response to an ACRDS is the one that gives the smallest response to a CRDS. This will often, but not always, have the opposite sign of disparity for the two types of stimulus.

Psychophysical studies have attempted to assess how the perception of depth from ACRDS can be linked to these neural responses. Computational models such as the binocular energy model allow us to predict the effects of anticorrelation on the encoding of binocular information, as a result of the inversion of disparity tuning functions. These individual neural responses, at the earliest stage of processing, are not in themselves sufficient to elicit the perception of depth. Rather, processing at later stages of the visual hierarchy, and subsequent feedback to these early stages, also play a role in the perception of depth. Understanding the perceptual consequences of the inversion of disparity tuning functions of early-stage binocular filters in ACRDS informs our understanding of these later stages of binocular processing.

When both the target and background in a random dot stimulus are anticorrelated, no depth perception is found even after extensive training [11]. When the target is anticorrelated and the background correlated, several studies have found that depth is perceived in the reversed direction [12–15], although others have found little evidence for reliable depth perception in similar stimuli, or that reversed depth may be reported by only a minority of participants [16, 17]. This reversed depth also appears to be possible only for simple near/far judgments, but not the perception of more complex structure [13]. When stimuli contained just a step edge in depth, the majority (67%) of participants who perceived depth in CRDS also perceived depth in the natural rather than reversed direction for ACRDS [17]. Finally, reversed depth in ACRDS has been reported to be more common when stimuli are presented in the periphery of the retinal image, rather than the centre [18, 19].

The primary purpose of the current study was to assess the combined effects of stimulus configuration and eccentricity on the perception of depth for ACRDS. Each of these stimulus properties has been shown to affect whether depth is perceived in ACRDS, and if so whether this is in the natural or reversed direction. Zhaoping and Ackerman [18] found that, for circular targets surrounded by an annulus, depth was seen in the reverse direction for peripheral stimuli, but not at all for centrally presented stimuli. Asher and Hibbard [17] found that, for stimuli presented at an eccentricity of 5.5 degrees, depth was seen in the natural direction for circular targets surrounded by a reference annulus, but in the natural direction for abutting rectangular target and reference stimuli. We were particularly interested in understanding how eccentricity would affect the perception of depth in these two stimulus configurations. These findings have also been used to propose models of how particular processing mechanisms, such as top-down feedback [20] or second-order channels [17], might contribute to the perception of depth.

The second purpose of the study was to assess the relationship between depth discrimination and perceptual confidence in ACRDS. While reliable depth perception in ACRDS has been reported in some studies, it is typically not accompanied by the clear, crisp, depth appearance found for equivalent CRDs stimuli [13]. We therefore expect that confidence might be low in ACRDS, even when performance differs from chance.

Finally, the third purpose of the study was to assess the importance of presentation time. Brief presentations will tend to enhance the responses of the transient stereoscopic system, which may be important in the perception of depth from ACRDS [21]. Longer durations will support the effects of top-down feedback, and thus the reduction of perceived depth in ACRDS [19, 20].

To address these three aims, we measured depth discrimination and confidence for CRDS and ACRDS. We varied the spatial configuration of the stimulus (a circular target with a surrounding annulus, or a simple horizontal depth edge); its eccentricity (between 0 and 12°); and the presentation duration (80ms or 700ms). For ACRDS stimuli defined by a single depth edge we show that, regardless of eccentricity or presentation time, depth tended to be perceived in the natural direction. In contrast, for the circular stimuli, when depth was seen it tended to be in the reversed direction.

## Experiment one

### Methods

#### Participants

3 participants (2 females, mean *(std)* age 34.5 *(9.6)*) completed the experiment, one (O1) was the co-author (PH), who had also served as a participant in a previous study [17] of the effects of shape on the perception of depth in ACRDS. Two students, who were naive to the aims of the study, were reimbursed for their participation. All had normal or corrected to normal vision, and stereoacuity of at least 50 arc sec, as measured using the Stereo Optical Butterfly Stereotest. All work was carried out in accordance with the Code of Ethics of the World Medical Association (Declaration of Helsinki). The study procedures were approved by the University of Essex Ethics Committee (Application No. JMA1901). All participants gave informed written consent.

#### Apparatus

Stimuli were presented on a VIEWPIXX 3D monitor, viewed from a distance of 50 cm. The monitor screen was 52 cm wide and 29 cm tall. The screen resolution was 1920 by 1080 pixels, with a refresh rate of 120 Hz. Each pixel subtended 1.9 arc min. Stereoscopic presentation was achieved using a 3DPixx IR emitter and NVIDIA 3D Vision LCD shutter glasses. The cross-talk between the left and right images, measured using a Minolta LS-110 photometer, was 0.12%. Participants’ responses were recorded using the computer keyboard. Stimuli were generated and presented using MATLAB and the Psychophysics Toolbox extensions.

#### Stimuli

**Fig 4:**
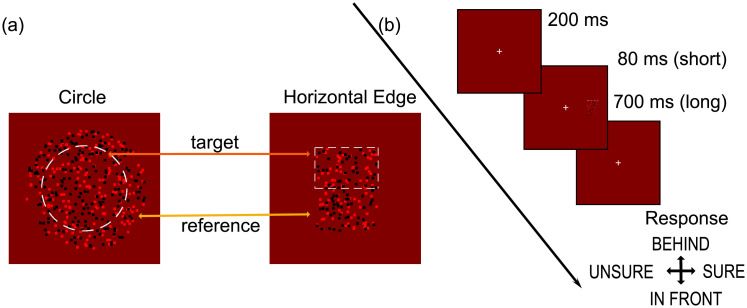
(a) Schematic of stimuli used for the Circle (left) and Horizontal Edge (right) conditions. For the circle condition the reference was the outside circle and for the horizontal edge condition the reference was always the bottom rectangle. The reference was always zero-disparity and the target disparity was 0 or ±28 arc minutes (where negative values are for uncrossed ‘further away’ and positive values are for crossed ‘closer’ targets). (b) Procedure: Targets (Circle or Horizontal Edge) were presented at one of 5 eccentricities (0°, 3.1°, 6.2°, 9.5° or 12.7°) for either 80ms or 700ms. Participants reported the position of the target (compared to the reference) making a response using the up arrow for a ‘further way’ or down arrow for a ‘closer’ response. This was immediately followed by a confidence judgment where right arrow was selected for ‘high confidence’, or left arrow for ‘low confidence/guessed’.

Stimuli in all conditions were random dot stereograms, consisting of 9 arc min square red (27.0 *cdm*^−2^) and black (0 *cdm*^−2^) dots against a red (13.5 *cdm*^−2^) background. Equal numbers of red and black dots were presented, with the number chosen so that they covered 25% of the stimulus area. In all cases, stimuli consisted of a correlated reference region, presented with 0 disparity, and a test region that was either correlated or anticorrelated. The dots were, in all cases, randomly repositioned on each frame. This created dynamic stimuli with a refresh rate of 60Hz. The test region was presented with disparities of -28, 0, or +28 arc min (where negative values signal uncrossed, far disparities, and positive values signal crossed, near disparities). In separate blocks of trials, stimuli were presented in the centre of the visual field, or to the right, so that the centre of the stimulus was at an eccentricity of 0°, 3.1°, 6.2°, 9.5° or 12.7°. Two stimulus configurations were used. The first was the circular condition, in which the target was a circular region with a radius of 2.3°. The reference was a surrounding annulus with an inner radius of 2.3° and an outer radius of 3.4°. The second was the horizontal edge condition, in which the reference and test regions were both a rectangle with a width of 3.40° and a height of 2.33°. The test region was vertically positioned directly above the horizontal midline, and the reference region directly below. The two stimulus configurations are illustrated in Fig 4(a).

#### Procedure

Each trial began with the presentation of a central fixation cross for 200 ms, followed by the presentation of a stimulus for either a short (80 ms) or long (700ms) duration. The fixation cross remained visible throughout each trial block. The combination of two stimulus configurations, and two presentation durations, resulted in four conditions. Each participant completed each condition within a single session. Within each condition, trials for each of the 5 eccentricities, and for correlated and anticorrelated stimuli, were presented in a separate block. In each block, each of the 3 disparities was presented 40 times. The order of presentation of these 10 blocks for each condition was randomised for each participant, as was the order of presentation of the four conditions.

After stimulus presentation, the participant was required to make two responses. The first was to indicate whether the target appeared closer (down arrow) or further away (up arrow) than the reference. The second was to indicate whether they felt confident in their response (right arrow) or that they were guessing (left arrow). The next trial began after the two responses had been made. See Fig 4(b).

### Results

**Fig 5:**
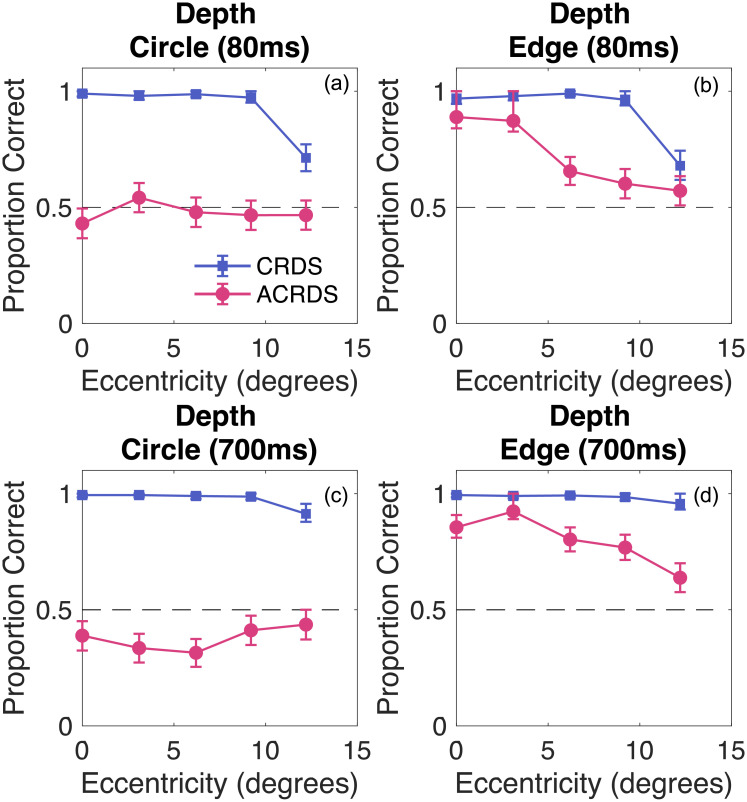
Proportion of correct depth responses as a function of eccentricity. The top row plots data for the short duration (80ms) for (a) circular and (b) horizontal edge stimuli. The bottom row plots data for the long duration (800ms) for (c) circular and (d) horizontal edge data. In all cases, the blue squares plot data for CRDS and the pink circles plot data for the ACRDS. Data show the mean results across participants, and bootstrapped 95 percent confidence limits. The horizontal dotted line shows chance performance. In all cases, depth was seen in the natural direction for CRDS. For the short duration ACRDS stimuli, there was no reliable perception of depth for the circular stimuli. Depth was perceived in the natural direction for edge stimuli, decreasing with eccentricity. For the long duration ACRDS stimuli, depth was seen in the reversed direction for circular stimuli, and in the natural direction for horizontal edge stimuli. For the edge stimuli, the reliability of perceived depth reduced with eccentricity.

**Fig 6:**
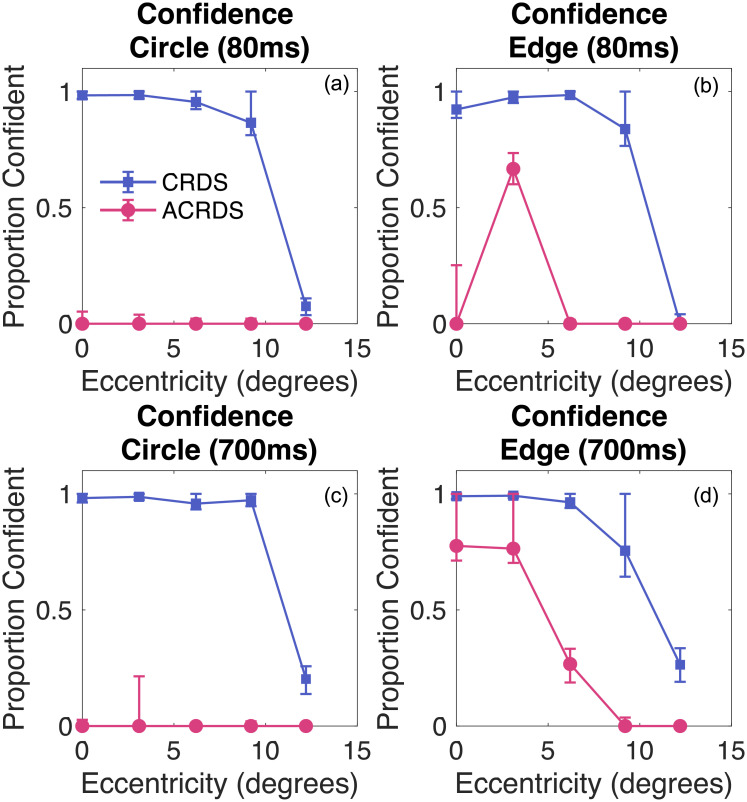
Proportion of confident responses as a function of eccentricity. The top row plots data for the short duration (80ms) for (a) circular and (b) horizontal edge stimuli. The bottom row plots data for the long duration (800ms) for (c) circular and (d) horizontal edge data. In all cases, the blue squares plot data for CRDS and the pink circles plot data for the ACRDS. Data show the mean results across participants, and bootstrapped 95 percent confidence limits. In all cases, confidence was high for centrally presented CRDS, and reduced with eccentricity. Confidence was generally low for ACRDS, with the exception of centrally presented edge stimuli at the long duration.

For each duration, and each stimulus type, the proportion of near responses, and the participant’s confidence, were recorded for CRDS and ACRDS. To evaluate performance on the depth task, we determined whether participants tended to consistently report natural or reversed depth, and whether this was affected by eccentricity. To do this, we combined data from the two non-zero disparities, coded for natural and reversed depth, irrespective of whether this was nearer or further than the reference. The average proportion across participants, as a function of eccentricity, is plotted in Fig 5 for each condition. Averages were obtained by calculating probit values from the proportion of correct responses, taking the mean of the probit values, then converting back to proportions. 95 percent confidence limits on these averages were derived using a bootstrap procedure with 10000 samples, in which the participants’ responses were resampled from a binomial distribution. These confidence limits allow us to evaluate whether, on average, depth judgments tended to be reliably above or below that expected by chance. The average proportions of confidence responses were calculated in the same way and are plotted in Fig 6. Data are plotted separately for each participant in the S1 File.

For each duration-by-shape-by-correlation condition, data were analysed with a generalised linear mixed effects model, with a binomial error function, a probit link function and eccentricity as the predictor. This allowed us to determine whether depth perception was in the natural or reversed direction, and how this was affected by eccentricity. In this model, a positive intercept indicates that depth tends to be perceived in the natural direction, and a negative intercept indicates that depth tends to be perceived in the reversed direction. A positive slope indicates an increasing number of natural depth responses with eccentricity, while a negative slope indicates a decreasing number of natural depth responses.

Our predictions regarding eccentricity were that, for the range of stimulus positions used, any effects of eccentricity would be monotonic. The range of eccentricities (up to 12.7 degrees) was chosen to be comparable to that used by Zhaoping and Ackerman [18], and the much larger disparities used in the current study mean that we would have expected reliable performance for a broader range of eccentricities than would have been the case with the small disparities used by Zhaoping and Ackerman. Eccentricity was thus included as a linear predictor.

#### Depth judgments

*Circular stimuli at short durations (80ms)*. For correlated stimuli, depth was perceived in the natural direction (*β*=5.12, t(13)=8.50, p<0.001), with the reliability of these judgments decreasing with eccentricity (*β*=-0.011, t(13)=-7.46, p<0.001) (Fig 5a). This indicates more variable responses at greater eccentricities [22–24]. For anticorrelated stimuli, depth judgments did not differ from chance (*β*=-0.052, t(13)=-0.685, p = 0.505), or vary with eccentricity (*β*=0.00, t(13)=-0.074, p = 0.942) (Fig 5a). We did not find an increased tendency to the perception of reversed depth with increasing eccentricity [18].

*Horizontal edge stimuli at short duration (80ms)*. For correlated stimuli, depth was perceived in the natural direction (*β*=5.89, t(13)=5.69, p<0.001), with the reliability of these judgments decreasing with eccentricity (*β*=-0.014, t(13)=-5.31, p<0.001) (Fig 5b). For anticorrelated edge stimuli, depth was perceived in the natural direction (*β*=0.916, t(13)=4.68, p<0.001), with the reliability of these judgments decreasing with eccentricity (*β*=-0.0020, t(13)=-2.94, p = 0.0116) (Fig 5b). Compared with the responses to anticorrelated circular stimuli, responses were reliable (overall 70% versus 47% correct responses).

*Circular stimuli at the long duration (700ms)*. For correlated stimuli, depth was perceived in the natural direction (*β*=4.61, t(13)=12.05, p<0.001), with the reliability of these judgments decreasing with eccentricity (*β*=-0.008, t(13)=-8.84, p<0.001) (Fig 5c). For anticorrelated stimuli, there was a tendency for depth to be perceived in the reverse direction (*β*=-0.391, t(13)=-3.68, p = 0.0028) (Fig 5c). This did not vary with eccentricity (*β*=0.0004, t(13)=1.10, p = 0.290).

*Horizontal edge stimuli at the long duration (700ms)*. For both correlated (*β*=3.35, t(13)=9.29, p<0.001) and anticorrelated (*β*=1.29, t(13)=8.26, p<0.001) stimuli, depth was perceived in the natural direction (Fig 5d). The reliability of these judgments decreased with eccentricity for both correlated (*β*=-0.0042, t(13)=4.5, p<0.001) and anticorrelated (*β*=-0.0022, t(13)=-4.077, p = 0.0013) stimuli.

#### Confidence judgments

*Circular stimuli at short durations (80ms)*. For correlated stimuli, confidence was high for centrally presented stimuli, and reduced with eccentricity. For anticorrelated stimuli,confidence was low, consistent with the low reliability of depth judgments (Fig 6a).

*Horizontal edge stimuli at short duration (80ms)*. For correlated edge stimuli, confidence was high for centrally presented stimuli, and reduced with eccentricity. For anticorrelated edge stimuli, confidence was low (Fig 6b).

*Circular stimuli at the long duration (700ms)*. For correlated stimuli, confidence was high for centrally presented stimuli, and reduced with eccentricity. Confidence was low for anticorrelated stimuli (Fig 6c).

*Horizontal edge stimuli at the long duration (700ms)*. For both correlated and anticorrelated stimuli, confidence was high for centrally presented stimuli, and reduced with eccentricity (Fig 6d).

### Discussion

The first experiment assessed the perception of depth, and participants’ confidence in their depth judgments, for correlated and anticorrelated random dot stereograms as a function of their horizontal eccentricity and presentation duration.

For correlated stimuli, in all cases accuracy and confidence were high for centrally presented stimuli and tended to reduce with eccentricity, as would be expected.

For anticorrelated stimuli, different results were found for the circular and horizontal edge stimuli. For circular stimuli, reversed depth was perceived at the long duration. For anticorrelated horizontal edge stimuli, in contrast, depth in the natural direction was perceived.

One complication in studying the effects of eccentricity on binocular stereopsis is the variation in binocularity, and stereoacuity, across the visual field. There are for example known differences in the horopter of corresponding points, and in the bias and precision of depth perception, between the upper and lower visual fields [25, 26]. These differences are reflected in the disparity tuning of cortical neurons [10, 26]. In the horizontal direction, stereoacuity also reduces with eccentricity [27], and the retinal blindspot limits binocular processing for eccentricities beyond around 12°, the maximum eccentricity used in the current study [28]. While the proximity of our stimuli to the right eye’s blindspot could have affected our results, we did not see a notable reduction in performance with CRDS at this eccentricity, over and above the general trend for reducing accuracy with increasing eccentricity. Nevertheless, the experiment was repeated for short duration stimuli at the same eccentricities in the vertical direction, to determine if there is a consistent effect of eccentricity on the direction of perceived depth, and to ensure that the findings at the furthest eccentricity were not affected by the influence of the blindspot.

## Experiment two

### Methods

**Fig 7:**
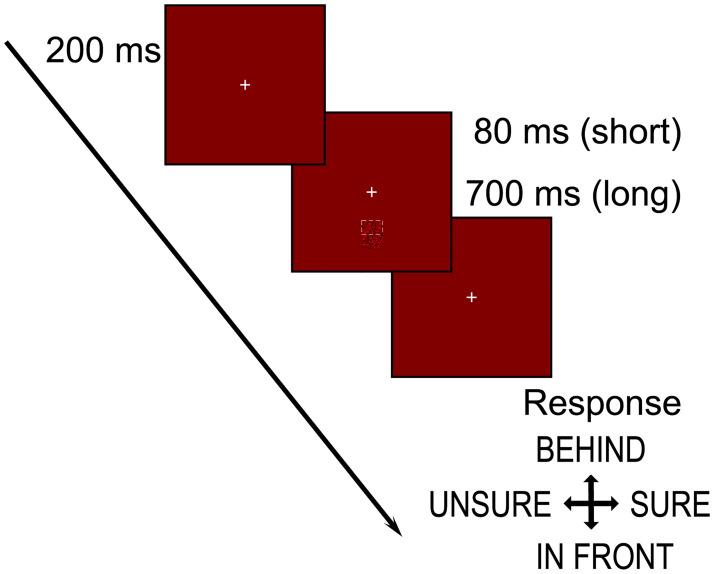
Targets (circle or horizontal edge) were presented below the fixation cross at one of 5 eccentricities (0°, 3.1°, 6.2°, 9.2° or 12.2°) for 80ms. Participants reported the position of the target (compared to the reference) making a response using the up arrow for a ‘further way’ or down arrow for a ‘closer’ response. This was immediately followed by a confidence judgment where right arrow was selected for ‘high confidence’, or left arrow for ‘low confidence/guessed’.

2 participants from Experiment 1 (O1 and O3) and a new participant O4 (naive to the aims) participated in this study. The apparatus were the same as in the first experiment. Horizontal edge stimuli were presented with the brief (80ms) duration, but in this experiment they were positioned below the central fixation, at eccentricities of (0°, 3.1°, 6.2°, 9.5° or 12.7°) (Fig 7).

### Results

**Fig 8:**
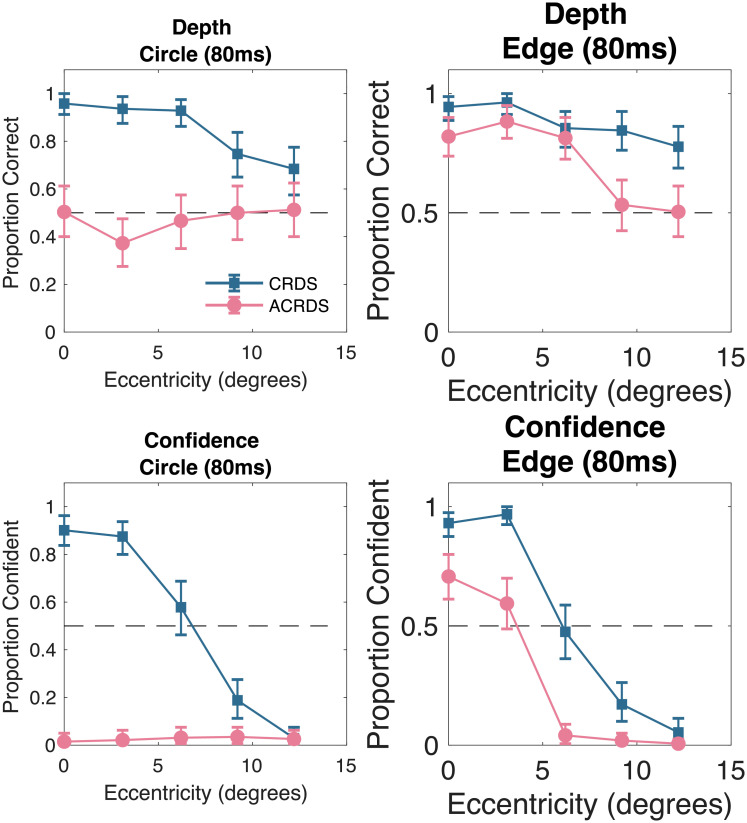
Accuracy and confidence of depth judgments for the short duration stimuli presented along the vertical meridian. The top row shows the accuracy judgments for the circular and edge stimuli, for each participant. The bottom row shows the confidence judgments. Error bars show 95 percent confidence intervals.

Results for the stimuli presented below fixation, on the vertical meridian, are summarised in (Fig 8).

#### Depth judgments

*Circular stimuli*. For correlated stimuli, participants perceived depth in the natural direction (*β*=1.50, t(13)=4.45, p<0.001), with the number of correct responses decreasing with eccentricity (*β*=-0.002, t(13)=-3.26, p = 0.006) (Fig 8a). For anticorrelated stimuli, there was no reliable trend for depth judgments in either the natural or reversed direction (*β*=-0.14, t(13)=-1.68, p = 0.118), and no effect of eccentricity (*β*=-0.0004, t(13)=1.067, p = 0.306)(Fig 8a).

*Horizontal edge stimuli*. For correlated stimuli, participants perceived depth in the natural direction (*β*=1.20, t(13)=3.35, p = 0.0052), and there was no reliable effect of eccentricity on accuracy (*β*=-0.0011, t(13)=-1.28, p = 0.222) Fig 8b). For anticorrelated stimuli, depth was perceived in the natural direction (*β*=0.89, t(13)=3.79, p = 0.00227), with the reliability of judgments decreasing with eccentricity (*β*=-0.0022, t(13)=-3.27, p = 0.00662) (Fig 8b).

#### Confidence judgments

*Circular stimuli*. For correlated stimuli, confidence was high for centrally presented stimuli, and reduced with eccentricity. For anticorrelated stimuli confidence was low for all eccentricities (Fig 8c).

*Horizontal edge stimuli*. For correlated stimuli, confidence was high for centrally presented stimuli, and reduced with eccentricity. For anticorrelated stimuli confidence was low for all eccentricities (Fig 8d).

### Discussion

The second experiment was performed to determine whether the eccentricity effects on the perception of depth were influenced by the presence of the blind spot, for the largest horizontal eccentricity tested. We found similar results when stimuli were presented below, rather than to the right of fixation. These results show that for stimuli presented on horizontal and vertical meridians, there was (i) a very limited tendency for perception of reversed depth for anticorrelated circular stimuli, (ii) natural depth perception for anticorrelated edge stimuli and (iii) an overall reduction in reliability and confidence with increasing eccentricity.

## General discussion

The current study assessed the effects of retinal eccentricity on the perception of depth from correlated and anticorrelated random dot stereograms, using both horizontal edge and embedded circular surface configurations. We replicated our previous finding of a striking difference in performance between these two stimuli, with unreliable reversed depth seen in some cases for circular stimuli (Fig 5b) and reliable, natural depth seen consistently for horizontal edge stimuli (Figs 5b and 5d and 8b).

For the horizontal edge stimuli, participants reliably reported depth in the natural direction in anticorrelated stimuli. Performance was more reliable when the presentation time was longer, and reduced with increasing eccentricity. For the circular stimuli, the perception of depth in anticorrelated stimuli was much weakened, but was in the reversed direction when it did occur, and again was more reliable at the longer duration. These results were found for stimuli on both the horizontal and the vertical meridians. Contrary to the prediction that reduced top-down feedback in the periphery would lead to increased perception of reversed depth [20], we found no such increase, and sustained perception of natural depth for simple edge stimuli. These results are not consistent with some previous findings [18].

In contrast to our findings, Zhaoping and Ackerman [18] found that reversed depth was reported more frequently when circular stimuli were presented in the periphery than in the centre. There were however a number of differences between the stimuli that might have contributed to this discrepancy. For example, Zhaoping and Ackerman used stimuli in which the dots in the display were replaced every 100ms, whereas our dot locations were updated every 16.7ms. We also used shutter glasses to present a different image to each eye. This means that the image presented to one eye was delayed by 8.3ms relative to the other, and the two images were not presented simultaneously. However, given the temporal integration of information in the visual system, this magnitude of delay is not expected to affect the perception of depth [13]. Our stimuli were also smaller, with the inner target region having a radius of 2.3 degrees, compared with the 3 degrees used by Zhaoping and Ackerman. The difference in refresh rate would have increased the number of samples in our stimuli and thus the information about the anticorrelation in ACRDS [29]. This might be expected to increase the strength of any depth signal. However, Hibbard et al. found no difference in performance between static and dynamic RDS, and no evidence for reversed depth, for larger (4.8 degree radius), centrally presented stimuli [16]. Doi et al. [14] reported reversed depth for dynamic, circular ACRDS with a radius of 2.5 degree, presented at an eccentricity of 3 degrees. The perception of reversed depth cannot therefore be attributed to the size, eccentricity, or temporal properties of the stimuli alone.

Another difference is the size of the disparity. Our disparity of 28 arc min was much larger than the 2.6, 5.1 and 10.2 arc min used by Zhaoping and Ackerman. Doi et al [14] argued that reversed depth in ACRDS is associated with coarse disparity processing mechanisms [30–33], and thus more likely to be found for large disparities. For stimuli at an eccentricity of 3 degrees, they found reversed depth from large (28.8 arc min) disparities, but no depth from small (1.8 arc min) disparities. The lack of reversed depth for centrally presented stimuli found by Zhaoping and Ackerman might thus be attributed to the use of a small disparity, in the range associated with fine disparity processing mechanisms.

A final difference is that Zhaoping and Ackerman used eye-tracking to ensure that fixation remained constant. This means that it is possible that, in our experiments, participants may have made eye-movements towards the stimuli, thus reducing their eccentricity. This would have been more likely for the long stimulus presentation. If so, then we might predict a smaller effect of eccentricity for the long stimulus presentation, and less tendency for the perception of reversed depth. We found a greater tendency for the perception of reversed depth with the longer duration, and no difference in the influence of eccentricity, which is not consistent with the predicted decrease in reversed depth if the effective eccentricity was reduced.

The effect of anticorrelation on the responses of binocular neurons in the primary visual cortex is to invert the disparity tuning function and to reduce the amplitude of response [34]. The perception of depth depends on the interpretation of these responses by later stages of processing. It is not thus possible to make simple predictions of how the inversion of disparity tuning will affect depth perception for ACRDS. However, several aspects of the overall response of binocular cells have been used to help explain the perception of depth in ACRDS.

The first reason for the lack of consistent and reliable depth perception in ACRDS is the inconsistency of responses across different binocular neurons. For each individual binocular neuron, the disparity tuning function depends not only on its position and phase disparity, but also on its orientation and spatial frequency tuning. So, for example, the peaks and troughs of this function will be closer together for a neuron tuned to a higher spatial frequency, and there will be more such peaks and troughs when the spatial frequency tuning bandwidth is narrower. For a correlated stimulus, the peaks in response will tend to occur for filters with the same preferred disparities across different scales, thus providing a consistent indication of the stimulus disparity. In contrast, the peaks in response for an anticorrelated stimulus will occur for filters with different preferred disparities, with the only consistency being a minimum in the expected response at the stimulus disparity. Across spatial scale, there is then no consistent evidence in support of the presence of a stimulus with a particular disparity [16, 17]. The sign and magnitude of depth signalled in ACRDS by individual neurons depends on the antipreferred disparity of the neurons, and there will be no single disparity that is consistent with the responses of the population as a whole.

The second reason why depth may not be perceived in ACRDS is that it is more closely associated with the response of binocular neurons at higher levels of cortical processing, where binocular matching is solved. For example, in area V4 the responses of some disparity-tuned cells to ACRDS do not depend on disparity, consistent with the general lack of reliable depth perception in these stimuli [35]. This has been taken as evidence that it is these higher levels of processing that provide the neural correlate of the perception of depth, rather than the disparity-tuned responses of neurons in V1.

**Fig 9:**
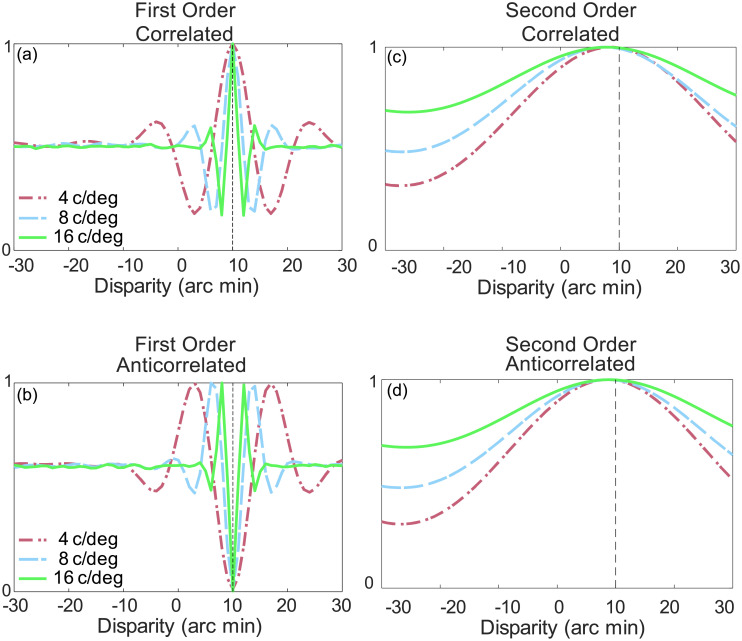
Left: Disparity tuning for first order mechanisms where the spatial frequency tunings of the filters were set to 4, 8 or 16 cycles/degree for (a) CRDS and (b) ACRDS. As predicted by the first order binocular energy response there is a peak at the correct disparity for CRDS (10 arc min), but an inverted peak for the preferred disparity for ACRDS. Right: Disparity tuning for second order mechanisms. Here the mean responses of the second order energy response model show a peak at the correct disparity for (c) CRDS (10 arc min), however for (d) the ACRDS, the inversion of the disparity tuning function is absent. For ACRDS second order models predict depth in the natural direction. Images reproduced with permission [17].

In cat area 18, the responses of some neurons are also consistent with the second-order disparity-processing channel [36], in which disparity can be defined by differences in contrast, rather than the underlying texture [37–40]. In the secon-order channel, the binocular energy calculation is preceded by an earlier stage of linear filtering, at a higher spatial frequency, and a rectifying nonlinearity (Fig 2) which makes the response insensitive to the contrast polarity of individual features (Fig 9). This sequence of calculations also means that strong matches can be achieved when the underlying random dot pattern is not matched between the left and right eyes’ image [38].

We have previously argued that the responses of the second-order channel might also explain the perception of depth in the natural direction that is found only for ACRDS stimuli defined by a single depth edge [17]. Since the second-order channel only signals simple depth [41], we proposed that it was not able to discriminate disparity in the stimuli in which the circular target was surrounded by an annulus.

While second-order stimuli support the perception of motion and depth, several studies have proposed that their contribution to figure-ground segmentation is limited. In the case of motion, Cavanagh and Mather [42] created random texture stimuli in which the motion of a central square region and the background, both defined by second-order cues, were in opposite directions. Participants could see the relative motion between the two areas, but there was no apparent segmentation between the two, and no impression of a subjective square. Dosher et al. [43] showed that the accuracy of reporting the direction of motion of a central square region in a random dot kinematogram, against a dynamic noise background, was greatly reduced when dots alternated in polarity. The perceived direction in this case depends on the balance between the contributions of the first- and second-order mechanisms, which is influenced by factors including the number of frames and the spatial scale of the stimulus [43–45]. These stimuli were also used to assess the strength of motion segmentation on the basis of second-order motion. In this case, participants were presented with a 3x3 grid of patches of planar motion. Eight of the patches moved in the same direction, while a randomly-selected target region moved in the opposite direction. Participants were not able to locate the target better than chance. It was concluded that second-order information supports the analysis of the direction of motion at only one or two locations in the image. The accurate perception of the direction of motion, but inability to use this information to segment an image into figure and ground, has also been found in the case of equiluminant [46] and dichoptic [47] motion.

In the case of binocular disparity, Ziegler and Hess showed that second-order stimuli support the perception of depth, but not three-dimensional shape or segmentation [41]. They showed that participants were unable to report the orientation of a depth corrugation defined by second-order disparity, even though they could accurately discriminate the depth of an individual element relative to fixation. They also investigated the perception of shape using the most elementary configuration, a step edge in depth. They showed that participants could accurately judge relative depth between two elements, but that performance rapidly reduced when additional elements were added. In contrast, participants were able to judge the overall depth, relative to fixation, when all elements had the same disparity. Second-order random noise stimuli have also been found to be unable to support the perception of depth from disparity when the noise is uncorrelated between the two eyes, providing a noisy first-order disparity signal [48]. When first-order disparities were set at zero, and second-order disparities signalled a sinusoidal depth corrugation, observers were able to distinguish this surface from a stimulus with a random distribution of disparities, but only if the corrugation had a large amplitude. In this case, observers perceived a step-like change in depth, rather than the smooth surface defined by the second-order disparities.

For both motion and binocular disparity, second-order, non-Fourier information is not effective in tasks requiring the comparison of information across multiple spatial locations [41, 43]. This is consistent with the idea that these mechanisms operate most effectively at large spatial scales [45].

We would thus expect the contribution of second-order mechanisms to the perception of three-dimensional shape to be very limited. In the case of our horizontal-edge stimuli, which contain many elements, we might predict that our participants would not accurately judge relative depth, based on previous results of Ziegler and Hess [41]. There are two important differences between our stimuli and theirs, however. The first is that one side of the step-edge was correlated in our stimuli, thus providing a robust first-order disparity signal. The second is that this reference region was presented at zero disparity in our stimuli, whereas Ziegler and Hess used stimuli with equal and opposite disparities either side of the edge. This means that averaging the disparity across the entire stimulus in our experiments would have provided a signal that would support the reliable perception of depth in the natural direction, as we found. These were necessary stimulus differences for two reasons. Firstly, a correlated reference stimulus was used since depth is not seen when an anticorrelated stimulus is presented against an anticorrelated background [12]. Secondly, this reference region was given a zero disparity, since presenting it with the opposite direction of disparity from that in the target region would have provided an unambiguous, correlated signal to the direction of depth without participants having to process the binocular information in the uncorrelated region.

If our participants were able to report the average disparity in this way, then we would also have expected accurate perception of depth for the circular stimuli. It would therefore have been possible for participants to perceive natural depth in both of our stimulus configurations from second-order mechanisms, even given the latter’s limited spatial resolution. That participants did not reliably perceive depth in the natural direction is consistent with their judgments being based on the relative depth between the centre and surround, and the poor spatial resolution in the second-order mechanism in providing this relative depth information.

One difference between our edge and circular stimuli is that the edge stimulus contains only a horizontal depth edge, while the circle contains edges at all orientations. A possible explanation for the difference in performance for the configurations might then be that second-order mechanisms are more sensitive to horizontal edges, reflecting the well-established stereoscopic anisotropy [49–54]. However, the perception of depth in the natural direction in step-edge stimuli does not appear to depend on orientation, since we have previously reported similar results for stimuli with a vertical edge [17].

Another reason why depth is not routinely perceived in ACRDS may be the role of top-down feedback. Perceptual inference has been characterised as a hierarchical process of analysis-by-synthesis, in which feedforward signals from V1 generate a perceptual hypothesis in higher visual areas [20]. In the case of binocular disparity this is the estimation of depth based on the disparity-tuned inputs from V1. The hypothesised binocular disparity is then used to synthesise a putative visual input, which is fed back to V1 for comparison with the actual input. A good match between the actual and hypothesised inputs then strengthens the support for the hypothesis, while a poor match will weaken the support. A naturally-viewed surface will create an input that is correlated across the two eyes. In the case of ACRDS, there will thus be a mismatch between the predicted positive correlation, and the actual negative correlation in the stimulus. It is this mismatch that is proposed to account for the lack of perceived depth. It has been suggested that this feedback process is weaker in the periphery than in central vision [20]. This reflects the distinct roles played by peripheral and central vision in input selection and decoding, respectively. It is also consistent with physiological evidence that the periphery is less well represented in higher cortical areas such as V3, V4 and V5/MT [55, 56]. The reduced higher-level representation of the peripheral visual field reduces the opportunity for feedback to V1, and thus for the depth suggested by the disparity-tuned responses to ACRDS to be vetoed [18]. It is the lack of this top-down feedback that is argued to allow for reversed depth to be perceived from peripherally presented ACRDS, based on the responses in V1. For centrally presented stimuli, the evidence for the hypothesised depth is down-weighted, meaning that depth is not perceived. This difference in feedback was proposed to account for the effect of eccentricity on the perception of depth in ACRDS reported by [18]. The lack of feedback in itself is not proposed to account for the reversal of depth, which is assumed to result from the inversion of the disparity tuning functions of binocular neurons in V1.

Anticorrelated stimulus elements can also contribute to strengthening the perception of depth in central vision, when combined with correlated elements with the opposite disparity [19]. This study also showed that anticorrelated elements disrupted the perception of depth from correlated depth when presented briefly, and with the same disparity. This disruption was not found for longer stimulus presentations. This was attributed to the vetoing of reversed-depth signals from anticorrelated elements by top-down feedback, when sufficient time was available for this to occur. This account again relied on the assumption that the depth signalled by first-order mechanisms would be in the reverse direction.

The location of stimuli in the visual field is also expected to affect the contributions of first- and second-order mechanisms to the perception of depth. Second-order stimuli can be detected in the periphery, and sensitivity loss with eccentricity scales similarly for first- and second-order stimuli [57–60]. However, the manner in which these channels are combined differs between central and peripheral vision [61]. In central vision, first- and second-order channels can be accessed separately. In contrast, perception in the periphery is subjected to ‘feature-blur’, such that multiple features, including the responses of first- and second-order channels, are blended together. This pooling of information across first- and second-order channels has also been proposed as a way to improve the accuracy of disparity estimation [62].

When applied to ACRDS, the first-order channel may be assumed to signal depth in the natural or reversed direction, and the second-order channel to signal depth in the natural direction. This may result in perceptual transparency [39, 40] or bistability [63, 64]. Given the potential difference in direction and magnitude signalled by the two channels, and in the reliability of this information, it is not possible to predict unequivocally whether this will result in an overall tendency to perceive depth in the reversed or natural direction, or how this would vary with eccentricity. This is true regardless of whether the channels are accessed independently or combined.

Studies that have found reversed depth in ACRDS have often used brief presentation times. Depth perception in opposite-polarity stimuli has been proposed to depend on the transient system, which relies on low spatial frequency components, and is not able to process spatially complex stimuli [65]. From this, we would predict that natural depth from second-order signals would be stronger for brief rather than long presentations. Additionally, feedback signals are predicted to be stronger following longer stimulus presentations [20], leading to a reduction of any signals for reversed depth in ACRDS in comparison with short presentations, if the perception of depth is moderated by feedback signals. Consistent with this prediction, the disruptive effects of uncorrelated elements on the perception of depth from correlated elements was found to reduce with increasing presentation time [19]. Increasing the presentation duration enhanced depth in the natural direction (for edge stimuli) and in the reverse direction (for circular stimuli). However, the extent of the effect did not change with increased eccentricity.

In conclusion, we confirm a striking difference in the perception of depth from ACRDS between horizontal edge stimuli, which tend to be perceived in the natural direction, consistent with the binocular disparity, and stimuli in which a circular target is surround by a reference annulus, which tend to be perceived in the reversed direction. This result was consistent for stimuli with different presentation times and eccentricities, and when presented along the horizontal and vertical meridians.

## Supporting information

S1 File Plots of individual data for all observers. (PDF)

## References

[1] Poggio GF, Gonzalez F, Krause F. Stereoscopic mechanisms in monkey visual cortex: Binocular correlation and disparity selectivity. Journal of Neuroscience. 1988;8(12):4531–4550. doi: 10.1523/JNEUROSCI.08-12-04531.1988 3199191 PMC6569547

[2] Hibbard PB. Binocular energy responses to natural images. Vision Research. 2008;48(12):1427–1439. doi: 10.1016/j.visres.2008.03.013 18456305

[3] Parker AJ. Binocular depth perception and the cerebral cortex. Nature Reviews Neuroscience. 2007;8(5):379–91. doi: 10.1038/nrn2131 17453018

[4] Ohzawa I, DeAngelis GC, Freeman RD. Stereoscopic depth discrimination in the visual cortex: Neurons ideally suited as disparity detectors. Science. 1990;249(4972):1037–1041. doi: 10.1126/science.2396096 2396096

[5] Fleet DJ, Wagner H, Heeger DJ. Neural Encoding of Binocular Disparity: Energy Models, Position Shifts and Phase Shifts. Vision Research. 1996;36(12):1839–1857. doi: 10.1016/0042-6989(95)00313-4 8759452

[6] Julesz B. Foundations of cyclopean perception. U. Chicago Press; 1971.

[7] Anzai A, Ohzawa I, Freeman RD. Neural mechanisms for encoding binocular disparity: receptive field position versus phase. Journal of Neurophysiology. 1999;82(2):874–890. doi: 10.1152/jn.1999.82.2.874 10444684

[8] Anzai A, Ohzawa I, Freeman RD. Neural mechanisms for processing binocular information I. Simple cells. Journal of neurophysiology. 1999;82(2):891–908. doi: 10.1152/jn.1999.82.2.891 10444685

[9] Anzai A, Ohzawa I, Freeman RD. Neural mechanisms for processing binocular information II. Complex cells. Journal of Neurophysiology. 1999;82(2):909–924. doi: 10.1152/jn.1999.82.2.891 10444686

[10] Prince SJD, Cumming BG, Parker AJ. Range and Mechanism of Encoding of Horizontal Disparity in Macaque V1. Journal of Neurophysiology. 2012;87(1):209–221. doi: 10.1152/jn.00466.2000 11784743

[11] Cumming BG, Shapiro SE, Parker AJ. Disparity detection in anticorrelated stereograms. Perception. 1998;27(11):1367–1377. doi: 10.1068/p271367 10505181

[12] Read JCAA, Eagle RA. Reversed stereo depth and motion direction with anti-correlated stimuli. Vision research. 2000;40(24):3345–3358. doi: 10.1016/S0042-6989(00)00182-6 11058733

[13] Tanabe S, Yasuoka S, Fujita I. Disparity-energy signals in perceived stereoscopic depth. Journal of vision. 2008;8(3):22. doi: 10.1167/8.3.22 18484828

[14] Doi T, Tanabe S, Fujita I. Matching and correlation computations in stereoscopic depth perception. Journal of Vision. 2011;11(3):1. doi: 10.1167/11.3.1 21367941

[15] Aoki SC, Shiozaki HM, Fujita I. A relative frame of reference underlies reversed depth perception in anticorrelated random-dot stereograms. Journal of Vision. 2017;17(12):17. doi: 10.1167/17.12.17 29071354

[16] Hibbard PB, Scott-Brown KKC, Haigh ECEC, Adrain M. Depth perception not found in human observers for static or dynamic anti-correlated random dot stereograms. PLoS ONE. 2014;9(1):e84087. doi: 10.1371/journal.pone.0084087 24416195 PMC3885516

[17] Asher JM, Hibbard PB. First- and second-order contributions to depth perception in anti-correlated random dot stereograms. Scientific Reports. 2018;8(14120):1–20. doi: 10.1038/s41598-018-32500-4 30237535 PMC6148546

[18] Zhaoping L, Ackermann J. Reversed Depth in Anticorrelated Random-Dot Stereograms and the Central-Peripheral Difference in Visual Inference. Perception. 2018;47(5):531–539. doi: 10.1177/0301006618758571 29514559

[19] Zhaoping L. Contrast-reversed binocular dot-pairs in random-dot stereograms for depth perception in central visual field: Probing the dynamics of feedforward-feedback processes in visual inference. Vision Research. 2021;186:124–139. doi: 10.1016/j.visres.2021.03.005 34091397

[20] Zhaoping L. Feedback from higher to lower visual areas for visual recognition may be weaker in the periphery: Glimpses from the perception of brief dichoptic stimuli. Vision Research. 2017;136:32–49. doi: 10.1016/j.visres.2017.05.002 28545983

[21] Pope DR, Edwards M, Schor CS. Extraction of depth from opposite-contrast stimuli: transient system can, sustained system can’t. Vision Research. 1999;39(24):4010–4017. doi: 10.1016/S0042-6989(99)00106-6 10748934

[22] Rawlings SC, Shipley T. Stereoscopic acuity and horizontal angular distance from fixation. JOSA. 1969;59(8):991–993. doi: 10.1364/JOSA.59.000991 5802956

[23] McKee SP. The spatial requirements for fine stereoacuity. Vision research. 1983;23(2):191–198. doi: 10.1016/0042-6989(83)90142-6 6868394

[24] Wardle SG, Bex PJ, Cass J, Alais D. Stereoacuity in the periphery is limited by internal noise. Journal of Vision. 2012;12(6):1–12. doi: 10.1167/12.6.12 22685339 PMC4502945

[25] Hibbard PB, Bouzit S. Stereoscopic correspondence for ambiguous targets is affected by elevation and fixation distance. Spatial vision. 2005;18(4):399–411. doi: 10.1163/1568568054389589 16167773

[26] Sprague WW, Cooper EA, Tošić I, Banks MS. Stereopsis is adaptive for the natural environment. Science advances. 2015;1(4):e1400254. doi: 10.1126/sciadv.1400254 26207262 PMC4507831

[27] Shipley T, Popp M. Stereoscopic acuity and retinal eccentricity. Ophthalmic Research. 1972;3(4):251–255. doi: 10.1159/000266200

[28] Wolf E, Morandi AJ. Retinal sensitivity in the region of the blind spot. JOSA. 1962;52(7):806–812. doi: 10.1364/JOSA.52.000806 14007924

[29] Henriksen S, Cumming BG, Read JCAA. A Single Mechanism Can Account for Human Perception of Depth in Mixed Correlation Random Dot Stereograms. PLoS Computational Biology. 2016;12(5):1–21. doi: 10.1371/journal.pcbi.1004906 27196696 PMC4873186

[30] Ogle KN. Disparity limits of stereopsis. AMA archives of ophthalmology. 1952;48(1):50–60. doi: 10.1001/archopht.1952.00920010053008 14932562

[31] Norcia AM, Suiter EE, Tyler CW. Electrophysiological evidence for the existence of coarse and fine disparity mechanisms in human. Vision research. 1985;25(11):1603–1611. doi: 10.1016/0042-6989(85)90130-0 3832583

[32] Tyler CW. A stereoscopic view of visual processing streams. Vision research. 1990;30(11):1877–1895. doi: 10.1016/0042-6989(90)90165-H 2288096

[33] Wilcox LM, Allison RS. Coarse-fine dichotomies in human stereopsis. Vision research. 2009;49(22):2653–2665. doi: 10.1016/j.visres.2009.06.004 19520102

[34] Cumming BG, Parker AJ. Responses of primary visual cortical neurons to binocular disparity without depth perception. Nature. 1997;389(6648):280–283. doi: 10.1038/38487 9305841

[35] Tanabe S, Umeda K, Fujita I. Rejection of false matches for binocular correspondence in macaque visual cortical area V4. Journal of Neuroscience. 2004;24(37):8170–8180. doi: 10.1523/JNEUROSCI.5292-03.2004 15371518 PMC6729782

[36] Tanaka H, Ohzawa I. Neural basis for stereopsis from second-order contrast cues. Journal of Neuroscience. 2006;26(16):4370–4382. doi: 10.1523/JNEUROSCI.4379-05.2006 16624957 PMC6673996

[37] Hess RF, Wilcox LM. Linear and non-linear filtering in stereopsis. Vision research. 1994;34(18):2431–2438. doi: 10.1016/0042-6989(94)90287-9 7975282

[38] Wilcox LM, Hess RF. Is the site of non-linear filtering in stereopsis before or after binocular combination? Vision Research. 1996;36(3):391–399. doi: 10.1016/0042-6989(95)00110-7 8746228

[39] Langley K, Fleet DJDJ, Hibbard PB. Linear and nonlinear transparencies in binocular vision. Proceedings of the Royal Society of London B: Biological Sciences. 1998;265(1408):1837–1845. doi: 10.1098/rspb.1998.0510 9802240 PMC1689373

[40] Langley K, Fleet DJDJ, Hibbard PB. Stereopsis from contrast envelopes. Vision Research. 1999;39(14):2313–2324. doi: 10.1016/S0042-6989(98)00271-5 10367053

[41] Ziegler LR, Hess RF. Stereoscopic depth but not shape perception from second-order stimuli. Vision Research. 1999;39(8):1491–1507. doi: 10.1016/S0042-6989(98)00224-7 10343817

[42] Cavanagh P, Mather G. Motion: the long and short of it. Spatial vision. 1989;. 2487159 10.1163/156856889x00077

[43] Dosher BA, Landy MS, Sperling G. Kinetic depth effect and optic flow—I. 3D shape from Fourier motion. Vision research. 1989;29(12):1789–1813. doi: 10.1016/0042-6989(89)90161-2 2631400

[44] Anstis S. Phi movement as a subtraction process. Vision research. 1970;10(12):1411–IN5. doi: 10.1016/0042-6989(70)90092-1 5516541

[45] Chubb C, Sperling G. Two motion perception mechanisms revealed through distance-driven reversal of apparent motion. Proceedings of the National Academy of Sciences. 1989;86(8):2985–2989. doi: 10.1073/pnas.86.8.2985 16594030 PMC287045

[46] Cavanagh P, Boeglin J, Favreau OE. Perception of motion in equiluminous kinematograms. Perception. 1985;14(2):151–162. doi: 10.1068/p140151 4069945

[47] Carney T, Shadlen MN. Dichoptic activation of the early motion system. Vision Research. 1993;33(14):1977–1995. doi: 10.1016/0042-6989(93)90022-O 8249314

[48] Wilcox LM. First and second-order contributions to surface interpolation. Vision Research. 1999;39(14):2335–2347. doi: 10.1016/S0042-6989(98)00261-2 10367055

[49] Wallach H, Bacon J. Two forms of retinal disparity. Perception & Psychophysics. 1976;19(5):375–382. doi: 10.3758/BF03199396

[50] Bradshaw MF, Rogers BJ. Sensitivity to horizontal and vertical corrugations defined by binocular disparity. Vision research. 1999;39(18):3049–3056. doi: 10.1016/S0042-6989(99)00015-2 10664803

[51] Mitchison G, McKee S. Mechanisms underlying the anisotropy of stereoscopic tilt perception. Vision research. 1990;30(11):1781–1791. doi: 10.1016/0042-6989(90)90159-I 2288090

[52] Hibbard PB, Bradshaw MF, Langley K, Rogers BJ. The stereoscopic anisotropy: individual differences and underlying mechanisms. Journal of Experimental Psychology: Human Perception and Performance. 2002;28(2):469. 11999867 10.1037//0096-1523.28.2.469

[53] Bradshaw MF, Hibbard PB, Parton AD, Rose D, Langley K. Surface orientation, modulation frequency and the detection and perception of depth defined by binocular disparity and motion parallax. Vision Research. 2006;46(17):2636–2644. doi: 10.1016/j.visres.2006.02.011 16571356

[54] Serrano-Pedraza I, Read JC. Multiple channels for horizontal, but only one for vertical corrugations? A new look at the stereo anisotropy. Journal of Vision. 2010;10(12):10–10. doi: 10.1167/10.12.10 21047742

[55] Gattass R, Sousa APB, Gross CG. Visuotopic organization and extent of V3 and V4 of the macaque. Journal of Neuroscience. 1988;8(6):1831–1845. doi: 10.1523/JNEUROSCI.08-06-01831.1988 3385477 PMC6569322

[56] Fiorani M, Gattass R, Rosa MGP, Sousa APB. Visual area MT in the Cebus monkey: Location, visuotopic organization, and variability. Journal of Comparative Neurology. 1989;287(1):98–118. doi: 10.1002/cne.902870108 2794126

[57] Solomon JA, Sperling G. Full-wave and half-wave rectification in second-order motion perception. Vision Research. 1994;34(17):2239–2257. doi: 10.1016/0042-6989(94)90105-8 7941419

[58] Smith AT, Snowden RJ, Milne AB. Is global motion really based on spatial integration of local motion signals? Vision research. 1994;34(18):2425–2430. doi: 10.1016/0042-6989(94)90286-0 7975281

[59] Smith AT, Ledgeway T. Sensitivity to second-order motion as a function of drift temporal frequency and viewing eccentricity. Investigative Ophthalmology and Visual Science. 1997;38(4):403–410. 10.1016/s0042-6989(97)00134-x 9536363

[60] Vakrou C, Whitaker D, McGraw PV. Extrafoveal viewing reveals the nature of second-order human vision. Journal of Vision. 2007;7(14):1–15. doi: 10.1167/7.14.13 18217808

[61] Shapiro AG, Knight EJ, Lu ZL. A first- and second-order motion energy analysis of peripheral motion illusions leads to further evidence of “feature blur” in peripheral vision. PLoS ONE. 2011;6(4). doi: 10.1371/journal.pone.0018719 PMC3084698 21559513

[62] Hibbard PB, Goutcher R, Hunter DW. Encoding and estimation of first- and second-order binocular disparity in natural images. Vision Research. 2016;120:108–120. doi: 10.1016/j.visres.2015.10.016 26731646 PMC4802249

[63] Goutcher R, Hibbard PB. Evidence for relative disparity matching in the perception of an ambiguous stereogram. Journal of vision. 2010;10(12). doi: 10.1167/10.12.35 21047767

[64] Goutcher R, Hibbard PB. Mechanisms for similarity matching in disparity measurement. Frontiers in Psychology. 2014;4(JAN). doi: 10.3389/fpsyg.2013.01014 24409163 PMC3884144

[65] Edwards M, Pope DR, Schor CM. First- and second-order processing in transient stereopsis. Vision Research. 2000;40(19):2645–2651. doi: 10.1016/S0042-6989(00)00126-7 10958914

