# Supplementary material for: Robust natural depth for anticorrelated random dot stereogram for edge stimuli, but minimal reversed depth for embedded circular stimuli, irrespective of eccentricity
Source: PLoS One. 2022 Sep 22;17(9):e0274566. doi: 10.1371/journal.pone.0274566 (PMC9499282; doi:10.1371/journal.pone.0274566)
Supplement: S1 File — (PDF) [file pone.0274566.s001.pdf]

Supplementary Material: Robust natural depth for simple anticorrelated random dot stereogram stimuli, but minimal reversed depth for complex stimuli, irrespective of eccentricity

Paul B. Hibbard[1*], Jordi M. Asher[1]

1 Department of Psychology, University of Essex, Colchester, Essex, UK

*phibbard@essex.ac.uk

1 Experiment One: Stimuli presented along the horizontal meridian

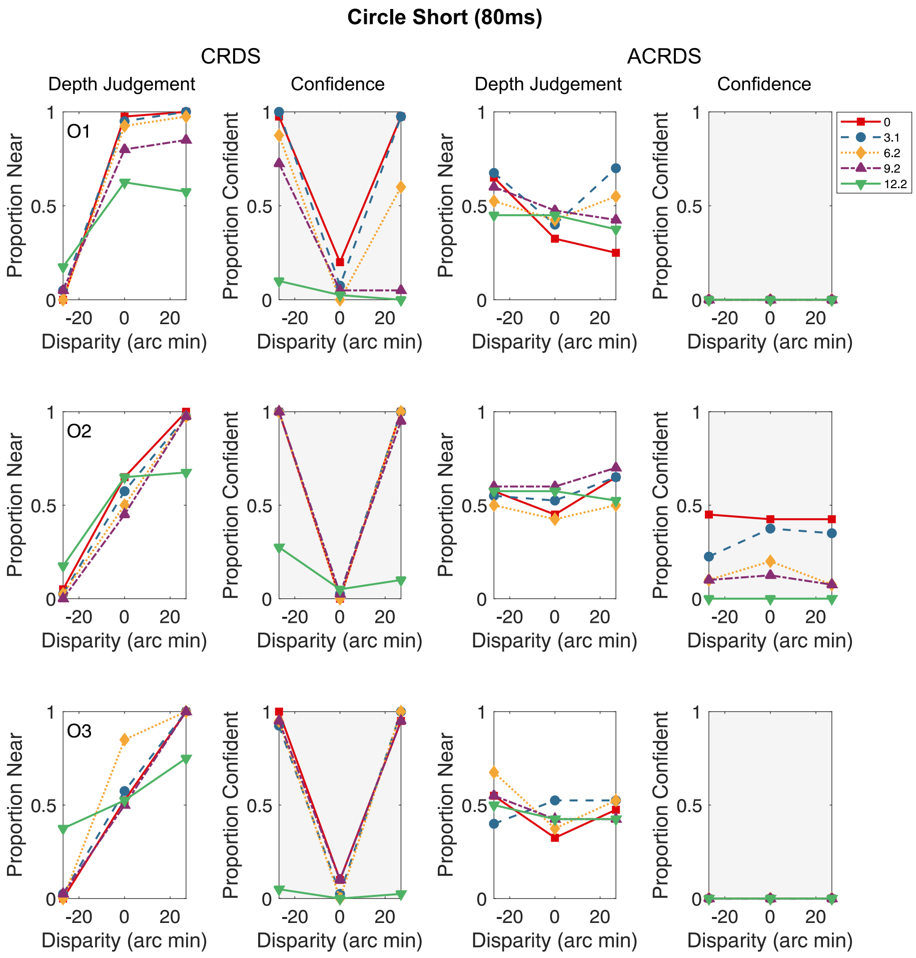

Fig 1. Raw data for circular short presentation (80ms) stimuli for each observer where responses from observer 1 (O1) are shown across the **top panels**, observer 2 (O2) in the **middle panels** and observer 3 (O3) along the **bottom panels**. Responses are sub-plotted for correlated stimuli (**left two panels**) and anticorrelated stimuli (**right two panels**). Depth responses are plotted as the proportion of near responses as a function of disparity where positive numbers show crossed (near) disparities. Each eccentricity (0°, 3.1°, 6.2°, 9.2°, 12.2°) is plotted as a different line. We also report the proportion of confident responses as a function of disparity. Each eccentricity is plotted as a line to match the depth response. For CRDS confidence is generally high at non-zero disparities with an exception for stimuli presented at 12.2°. Confidence was low for all ACRDS conditions. Error bars show 95 percent confidence intervals.

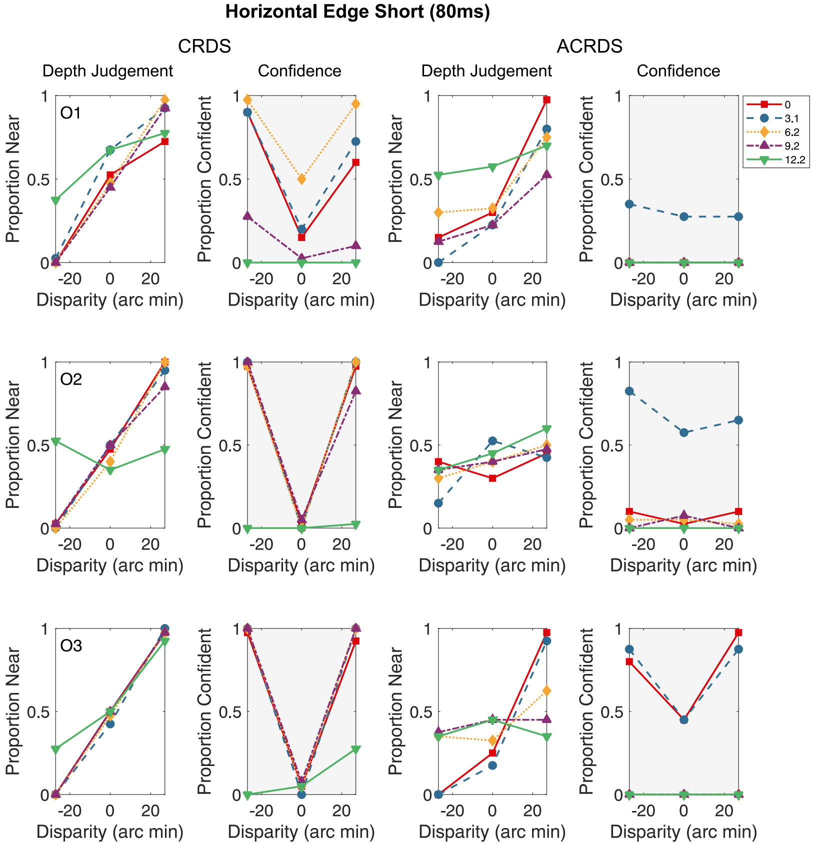

Fig 2. Raw data for horizontal edge short presentation (80ms) stimuli for each observer where responses from observer 1 (O1) are shown across the **top panels**, observer 2 (O2) in the **middle panels** and observer 3 (O3) along the **bottom panels**. Responses are sub-plotted for correlated stimuli (**left two panels**) and anticorrelated stimuli (**right two panels**). Depth responses are reported as the proportion of near responses and are plotted as a function of disparity where positive values represent crossed (near) disparities. Each eccentricity (0°, 3.1°, 6.2°, 9.2°, 12.2°) is plotted as a different line. We also report the proportion of confident responses as a function of disparity. As before, each eccentricity is plotted as a line to match the depth response. For CRDS confidence is generally high at non-zero disparities with ACRDS confidence showing individual variability between the observers. Error bars show 95 percent confidence intervals.

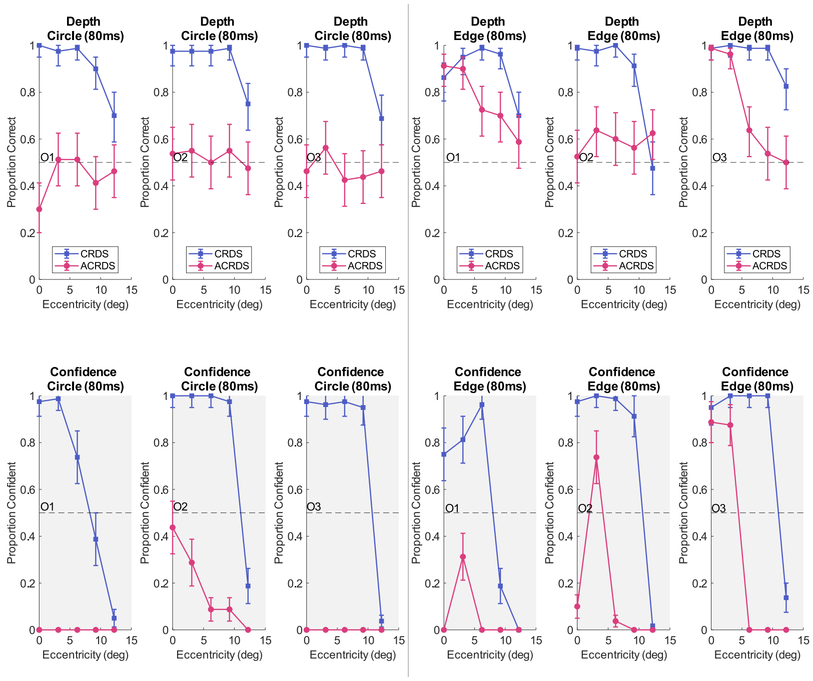

Fig 3. Accuracy and confidence of depth judgements for the short duration stimuli presented along the horizontal meridian. The top row shows the accuracy judgements for the circular and edge stimuli, for each observer. The bottom row shows the confidence judgements. Error bars show 95 percent confidence intervals.

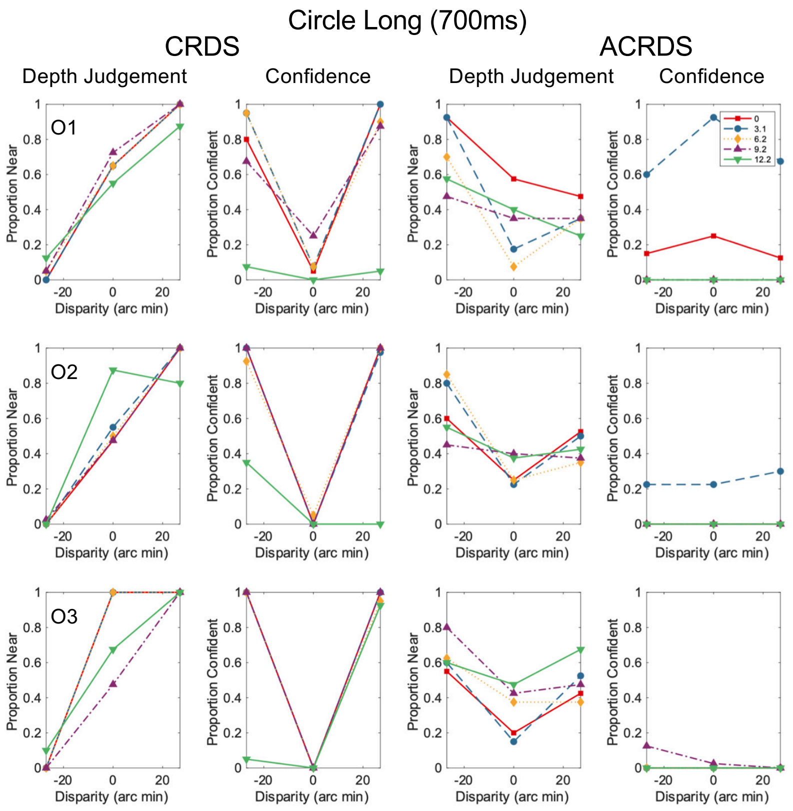

Fig 4. Raw data for circular long presentation (700ms) stimuli, plotted as for the short duration stimuli.

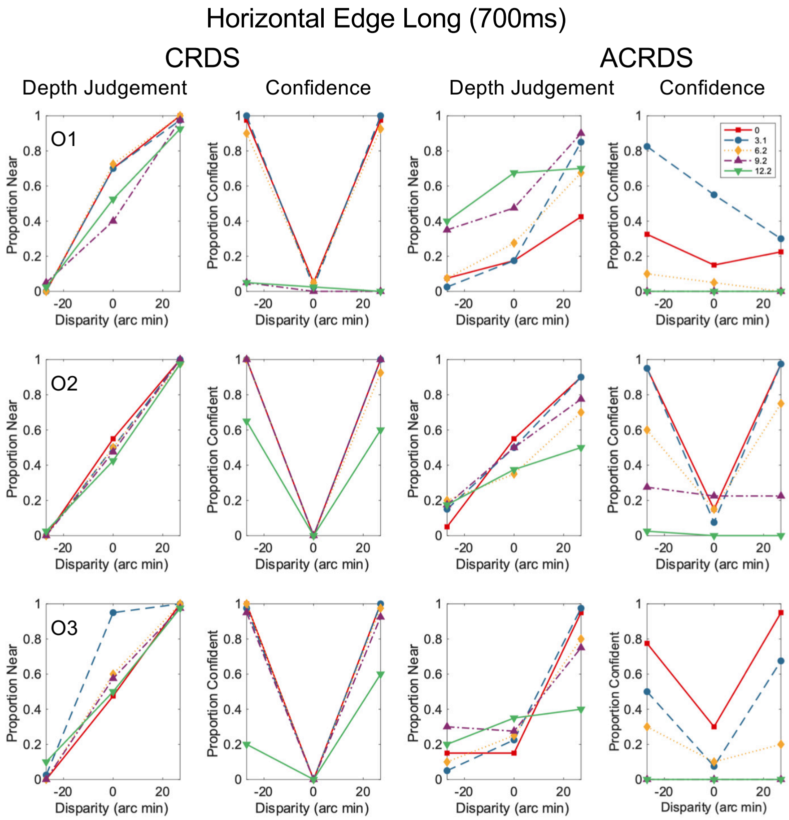

Fig 5. Raw data for circular long presentation (700ms) stimuli, plotted as for the short duration stimuli.

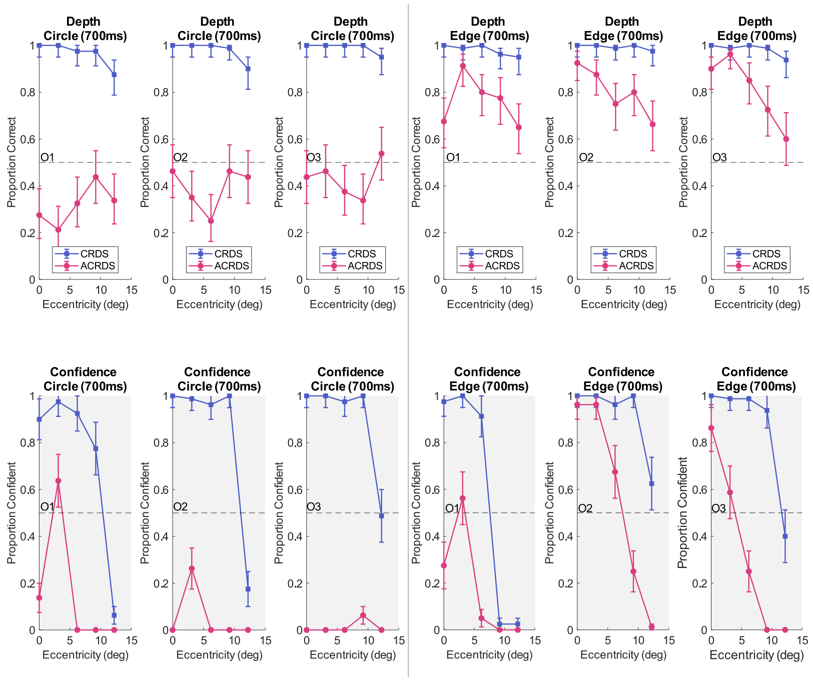

Fig 6. Accuracy and confidence of depth judgements for the long duration stimuli presented along the horizontal meridian. The top row shows the accuracy judgements for the circular and edge stimuli, for each observer. The bottom row shows the confidence judgements. Error bars show 95 percent confidence intervals.

2 Experiment Two: Stimuli presented along the vertical meridian

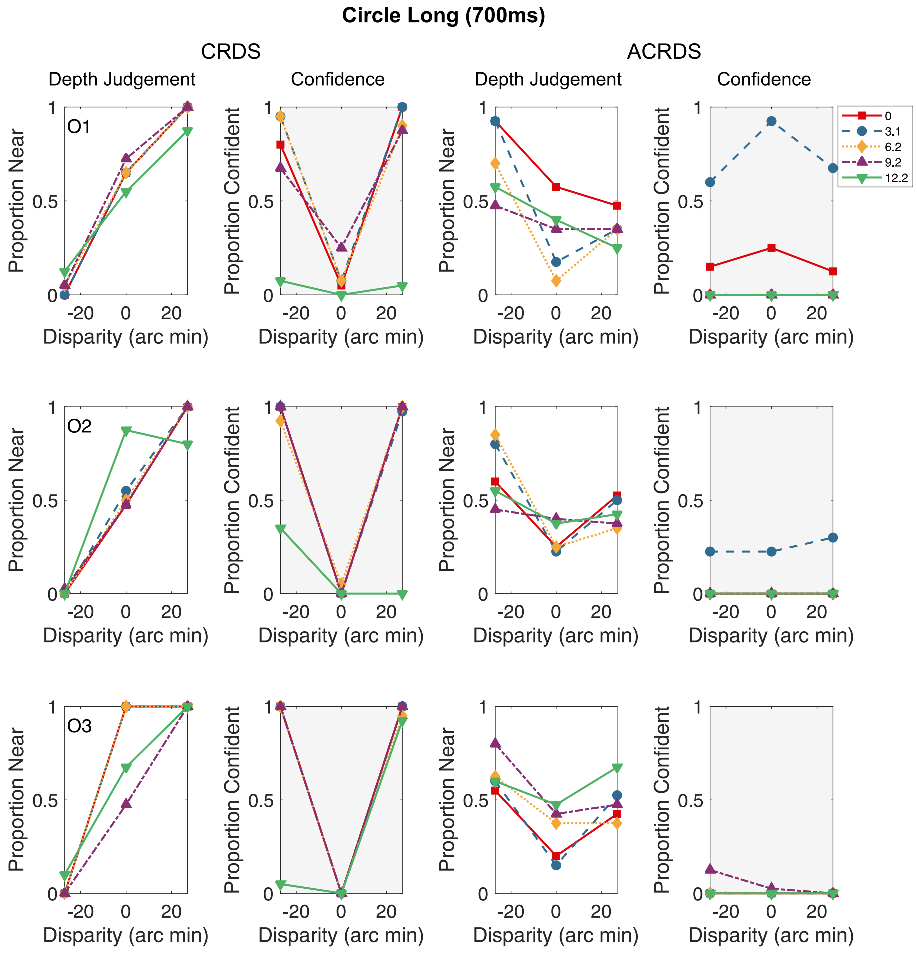

Fig 7. Raw data for the circular long presentation (700ms) stimuli for each observer where responses from observer 1 (O1) are shown across the **top panels**, observer 2 (O2) in the **middle panels** and observer 3 (O3) along the **bottom panels**. Responses are sub-plotted for correlated stimuli (**left two panels**) and anticorrelated stimuli (**right two panels**). Depth responses are reported as the proportion of near responses and are plotted as a function of disparity where positive values represent crossed (near) disparities. Each eccentricity (0°, 3.1°, 6.2°, 9.2°, 12.2°) is plotted as a different line. We also report the proportion of confident responses. As before, each eccentricity is plotted as a line to match the depth response. For CRDS confidence is generally high at non-zero disparities with ACRDS confidence showing individual variability between the observers.

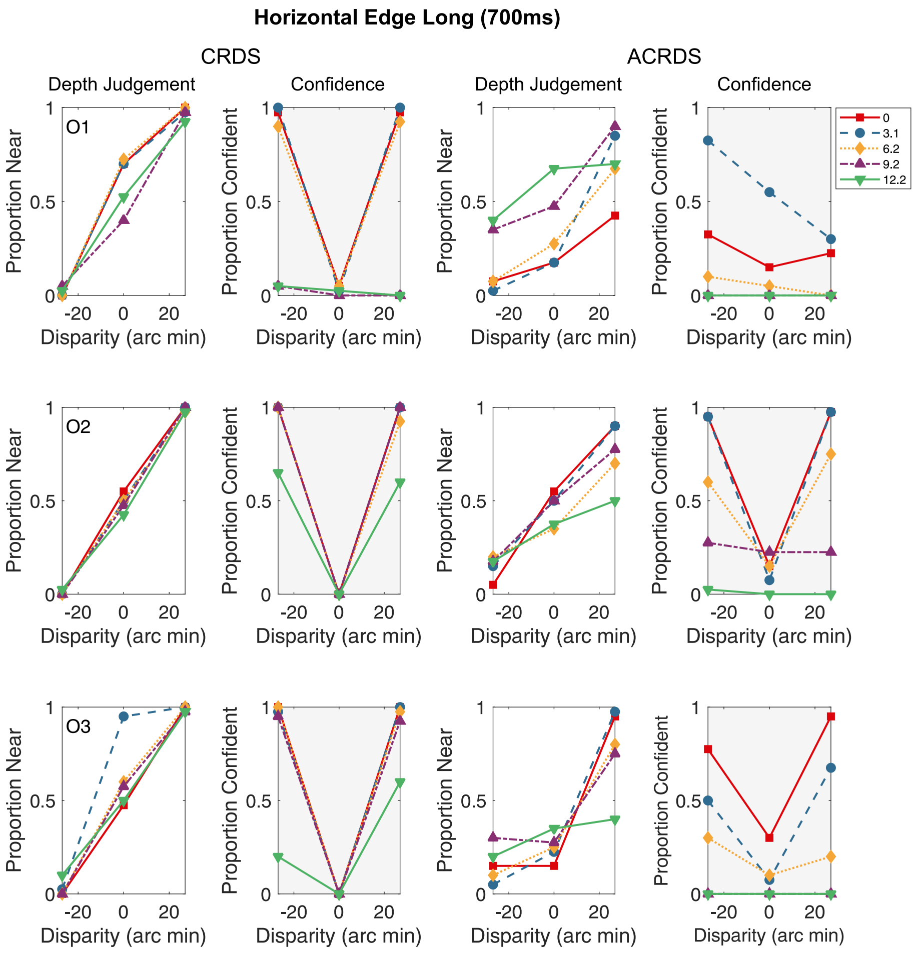

Fig 8. Raw data for the horizontal edge long presentation (700ms) stimuli for each observer where responses from observer 1 (O1) are shown across the **top panels**, observer 2 (O2) in the **middle panels** and observer 3 (O3) along the **bottom panels**. Responses are sub-plotted for correlated stimuli (**left two panels**) and anticorrelated stimuli (**right two panels**). Depth responses are reported as the proportion of near responses and are plotted as a function of disparity where 0 represents zero-disparity and negative numbers show a stimulus that is in front of the reference (zero-disparity). Each eccentricity (0°, 3.1°, 6.2°, 9.2°, 12.2°) is plotted as a different line. We also report confidence, which plotted is as a proportion (where 0 is low confidence and 1 is high confidence) as a function of disparity. As before each eccentricity is plotted as a line to match the depth response. For CRDS confidence is generally high at non-zero disparities with ACRDS confidence showing individual variability between the observers.

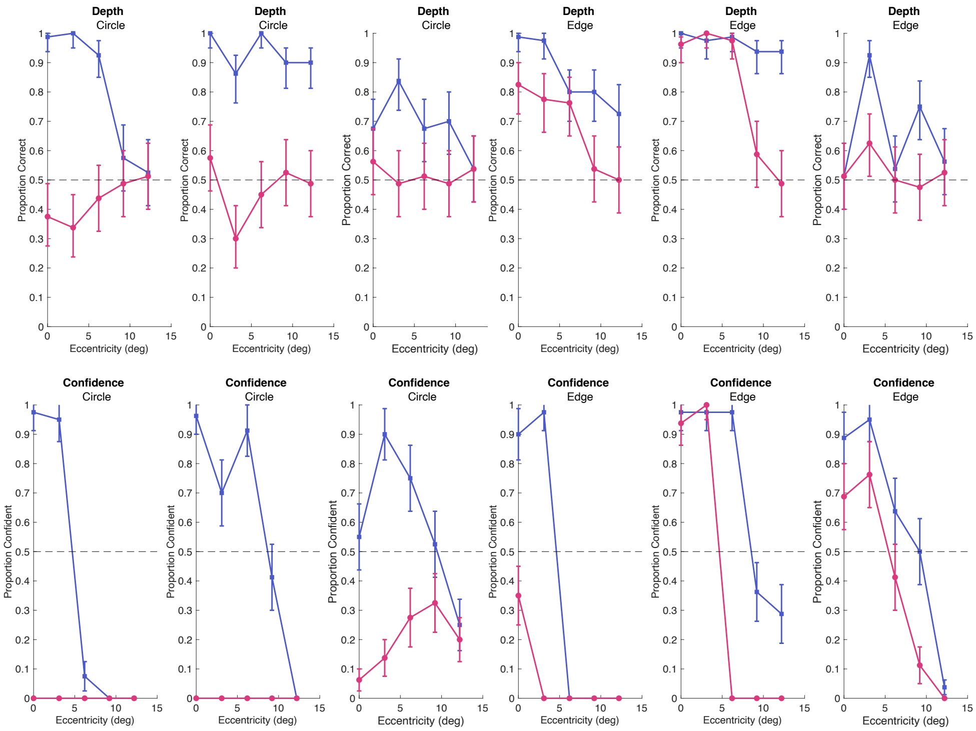

Fig 9. Accuracy and confidence of depth judgements for the long duration stimuli presented along the vertical meridian. The top row shows the accuracy judgements for the circular and edge stimuli, for each observer. The bottom row shows the confidence judgements. Error bars show 95 percent confidence intervals.
